# Plagiarism

**Published:** 1884-07

**Authors:** 


Plagiarism.—We regret to be obliged to allude to the disagreeable subject broached in the letter from Dr. Fleming, published in the present number. Dr. Fleming stands at the head of our profession, and merits all the honors which have been bestowed on him in consideration of the valuable work he has done.

That he has been guilty of the above weakness, however, Dr. Billings can prove, although in making the accusation Dr. Billings did not intend to say that the entire work of Franck had been translated literally, but only that portion of it which referred to the subject under consideration, viz: Eclamsia—a paper on which was read before the Gynecological Society of Boston, Mr. Fleming's doubt to the contrary notwithstanding.

